# Clinical and radiological findings of adult hospitalized patients with community-acquired pneumonia from SARS-CoV-2 and endemic human coronaviruses

**DOI:** 10.1371/journal.pone.0245547

**Published:** 2021-01-14

**Authors:** Moon Seong Baek, Min Jae Cha, Min-Chul Kim, Jin-Won Chung, Won-Young Kim, Hyewon Choi, Seong-Ho Choi

**Affiliations:** 1 Department of Internal Medicine, Chung-Ang University Hospital, Chung-Ang University College of Medicine, Seoul, Republic of Korea; 2 Department of Radiology, Chung-Ang University Hospital, Seoul, Republic of Korea; 3 Department of Infectious Diseases, Department of Internal Medicine, Chung-Ang University Hospital, Chung-Ang University College of Medicine, Seoul, Republic of Korea; National Institute for Infectious Diseases Lazzaro Spallanzani-IRCCS, ITALY

## Abstract

Endemic human coronaviruses (HCoVs) and severe acute respiratory syndrome coronavirus 2 (SARS-CoV-2) are members of the family *Coronaviridae*. Comparing the findings of the infections caused by these viruses would help reveal the novel characteristics of SARS-CoV-2 and provide insight into the unique pathogenesis of SARS-CoV-2 infection. This study aimed to compare the clinical and radiological characteristics of SARS-CoV-2 and endemic HCoVs infection in adult hospitalized patients with community-acquired pneumonia (CAP). This study was performed at a university-affiliated tertiary hospital in the Republic of Korea, between January 1, 2015, and July 31, 2020. A total of 109 consecutive patients who were over 18 years of age with confirmed SARS-CoV-2 and endemic HCoVs were enrolled. Finally, 19 patients with SARS-CoV-2 CAP were compared to 40 patients with endemic HCoV CAP. Flu-like symptoms such as cough, sore throat, headache, myalgia, and prolonged fever were more common in SARS-CoV-2 CAP, whereas clinical findings suggestive of bacterial pneumonia such as dyspnea, leukocytosis with left shift, and increased C-reactive protein were more common in endemic HCoV CAP. Bilateral peripherally distributed ground-glass opacities (GGOs) were typical radiologic findings in SARS-CoV-2 CAP, whereas mixed patterns of GGOs, consolidations, micronodules, and pleural effusion were observed in endemic HCoV CAP. Coinfection was not observed in patients with SARS-CoV-2 CAP, but was observed in more than half of the patients with endemic HCoV CAP. There were distinctive differences in the clinical and radiologic findings between SARS-CoV-2 and endemic HCoV CAP. Further investigations are required to elucidate the mechanism underlying this difference. Follow-up observations are needed to determine if the presentation of SARS-CoV-2 CAP changes with repeated infection.

## Introduction

At the end of 2019, severe acute respiratory syndrome coronavirus 2 (SARS-CoV-2) was discovered in Wuhan, China [1]. Coronavirus disease (COVID-19), the disease caused by SARS-CoV-2, can cause respiratory distress and severe pneumonia. COVID-19 has been the largest pandemic since the 1918 Spanish flu. The number of patients with severe pneumonia in Europe, the United States, and South America has paralyzed the respective healthcare systems, resulting in an explosive increase in the number of deaths mainly in vulnerable classes such as older adults or those with underlying disease [2]. To date, over 60 million COVID-19 cases and 1,400,000 deaths have been reported worldwide [3].

In the mid-1960s, two strains of human coronaviruses (HCoVs), 229E and OC43, were identified to be responsible for infections in humans. In 2004 and 2005, HCoV NL63 and HKU1 were discovered [4]. These four HCoVs are known to cause common colds and exhibit seasonal outbreaks every winter and spring. Thus, they are called endemic HCoVs [5]. Endemic HCoVs are found in 0.6%–2.5% of adults with community-acquired pneumonia (CAP) [5], and severe pneumonia caused by endemic HCoVs is occasionally reported in children, older adults, and immunocompromised hosts [6–8].

Although endemic HCoVs and SARS-CoV-2 belong to the family *Coronaviridae* and both may cause severe pneumonia, the patterns of their outbreaks are very different. Identifying the differences between these viruses would help reveal the novel characteristics of SARS-CoV-2 and provide insight into the unique pathogenesis of SARS-CoV-2 infection. To date, several clinical studies have compared SARS-CoV-2 infection with influenza or non-COVID-19 pneumonia. However, except for a study involving genetic differences, no clinical studies have compared SARS-CoV-2 with endemic HCoVs, especially in adult patients with CAP [9].

Therefore, we performed this study to compare the clinical and radiologic features of SARS-CoV-2 with endemic HCoVs in adult patients with CAP.

## Materials and methods

### Study design and patients

This study was performed at an 850-bed university-affiliated tertiary hospital in the Republic of Korea. The hospital included four nationally designated negative pressure isolation rooms, to which COVID-19 patients were hospitalized between February 1 and July 31, 2020. Consecutive patients diagnosed with endemic HCoVs between January 1, 2015, and July 31, 2020, were enrolled. We included patients who were ≥18 years old. We compared the clinical characteristics, laboratory findings, and radiologic findings between those with SARS-CoV-2 and endemic HCoV CAP.

The Institutional Review Board of Chung-Ang University Hospital approved this study and waived the need for patient consent due to the retrospective nature of the study (2009-004-19331).

### Data collection and definitions

The following data at admission were retrieved from the electronic medical records: demographic variables such as age and sex; body mass index; smoking status (current smoker, former smoker, or never smoked); symptom onset to hospital admission; initial symptoms; duration of fever; comorbidities; Charlson comorbidity index (CCI); CURB-65 (confusion, urea, respiratory rate, blood pressure plus age ≥ 65 years); modified early warning score (MEWS); sequential organ failure assessment (SOFA) score; vital signs including blood pressure, heart rate, and body temperature; treatment such as antibiotics, oxygen therapy, mechanical ventilation, vasopressor, and renal replacement therapy; laboratory findings; chest X-ray (CXR) or computed tomography (CT) findings; length of hospital stay; and in-hospital death.

### Diagnosis of SARS-CoV-2 and endemic HCoV pneumonia

To detect SARS-CoV-2 in the upper and lower respiratory tract specimens, the Allplex^TM^ 2019-CoV Assay (Seegene, Seoul, South Korea) was used [10]. For the detection of endemic HCoVs in nasopharyngeal swab specimens, an Allplex^TM^ respiratory panel (Seegene, Seoul, South Korea) was used, with a real-time polymerase chain reaction (PCR) thermocycler (Bio-Rad Laboratories, Inc., CA, USA). Automated ribonucleic acid extraction was carried out using the eMAG^TM^ system (bioMérieux, Inc., Marcy-I’Étoile, France).

Pneumonia was defined as the presence of a new or progressive infiltrate on a chest radiograph plus two or more of the following: fever, cough, sputum production, dyspnea, hemoptysis, and attending physician’s diagnosis of pneumonia. CAP was defined as described by Mandell LA, et al. [11]. If any respiratory pathogen was detected from the following tests within 48 h of admission, it was considered as a co-infecting pathogen, Gram stain/bacterial culture for blood and sputum, urinary antigen for *Streptococcus pneumoniae* and *Legionella pneumophila*, serological test for *Mycoplasma pneumoniae* and *Chlamydophila pneumoniae*, and the PCR-based methods for *C*. *pneumoniae*, *M*. *pneumoniae*, *L*. *pneumophila*, *Bordetella pertussis*, *and Bordetella parapertussis*.

### Image acquisition

Two thoracic radiologists (MJC and HC, with 11 and 6 years of experience in chest imaging interpretation, respectively) who were blinded to the clinical data independently reviewed all images in a random order. Decisions were made by consensus. The initial chest x-rays (CXRs) were classified as normal focal opacity and multifocal or diffuse opacity. The pattern of the disease course was also identified from serial CXRs, where type 1 was radiographic improvement, type 2 was radiographic deterioration by one peak level followed by improvement, type 3 was fluctuating radiographic changes with at least two peaks, and type 4 was progressive radiographic deterioration [12, 13]. On chest CT, the pattern of abnormalities was assessed for the presence of GGO, consolidation, and mixed pattern [14]. The presence of micronodules (<5 mm in diameter), pleural effusion, and lymphadenopathy were also recorded, and the laterality (unilateral vs. bilateral) of the lesions and the number of involved lobes (maximum five lobes) were evaluated. The distribution of parenchymal abnormalities was classified as central, peripheral, or mixed in the axial plane and upper, lower, or random in the longitudinal plane. Finally, CT scores ranging from 0 to 24 were calculated using a method described by Ooi et al. [15].

### Statistical analyses

Categorical variables are presented as numbers (%) and compared using Pearson’s Chi-square test or Fisher’s exact test. Continuous variables were expressed as the median (interquartile range) and compared with the Student’s *t* test or Mann-Whitney *U* test. Statistical significance was set at a *P*-value of < 0.05, and all statistical analyses were performed using the Package for the Social Sciences (SPSS), version 26.0 (IBM Corporation, Armonk, NY, USA).

## Results

**Fig 1:**
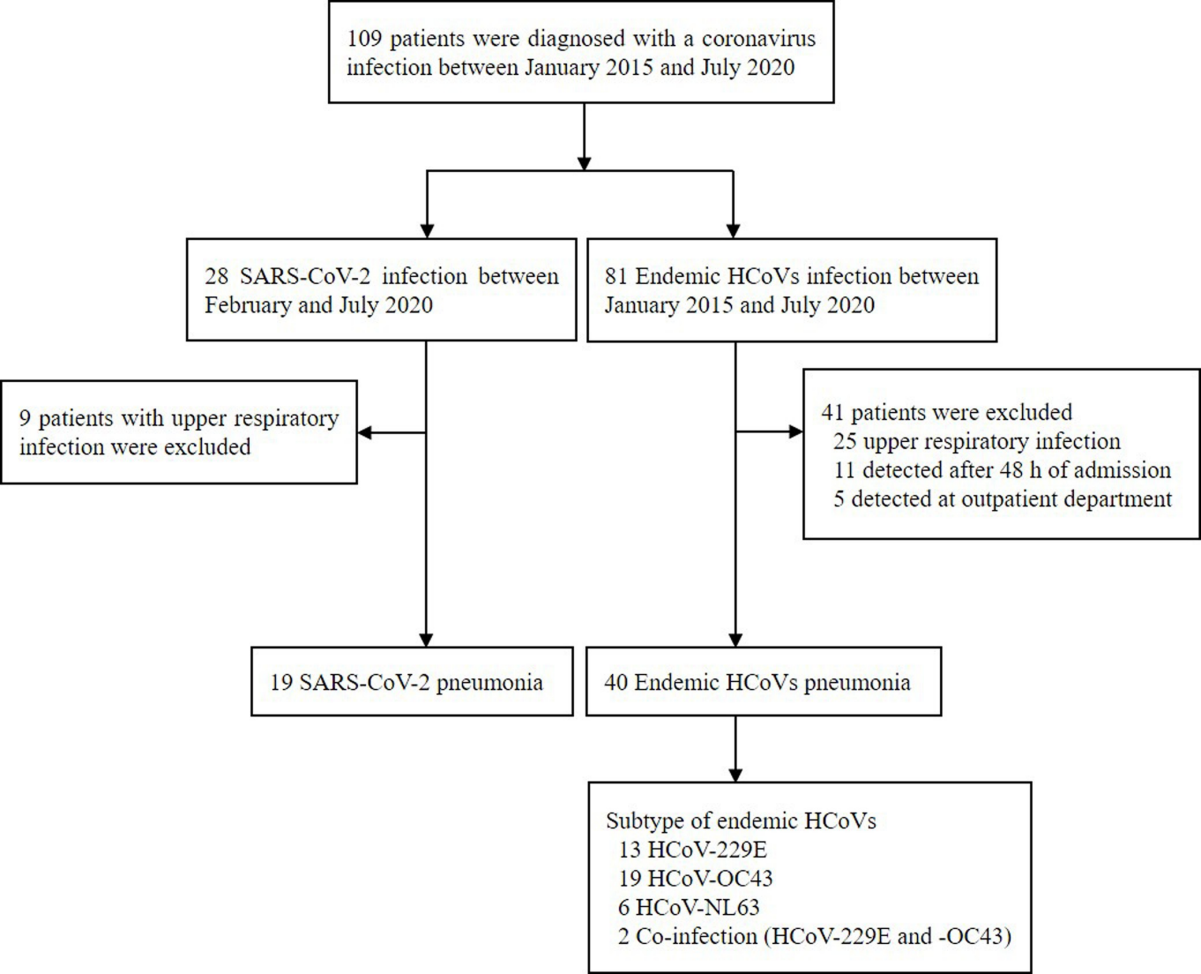
Flow chart of the patients. COVID-19, coronavirus disease; and HCoV, human coronavirus.

A total of 109 patients were diagnosed with CoV infection. Of these, 28 patients had SARS-CoV-2, and 81 had endemic HCoV pneumonia (Fig 1). Among them, data of 19 patients with SARS-CoV-2 pneumonia and 40 patients with endemic HCoV pneumonia were analyzed.

The baseline characteristics of the patients are shown in Table 1. The median age was 70 years (IQR, 62–78 years), and 32 (54.2%) patients were male. The most common symptom was fever (88.1%), lasting 2 days (IQR, 1–5 days). There was no significant difference in demographics such as age, sex, and body mass index between the two groups. Symptoms such as cough, sore throat, headache, and myalgia were common in patients with SARS-CoV-2 pneumonia. Symptom onset to hospital admission was longer in the SARS-CoV-2 group (7 days [IQR, 3–8 days] vs. 2 days [IQR, 0–5 d], p = 0.025). Duration of fever was longer in the SARS-CoV-2 group (4 d [IQR, 3–6 d] vs. 2 days [IQR, 1–4 d], p = 0.016). Notably, the patients with endemic HCoV pneumonia had a higher rate of dyspnea than those with SARS-CoV-2 pneumonia (15.8% vs. 47.5%, p = 0.019). Additionally, CCI, CURB-65, MEWS, and SOFA scores were higher in the endemic HCoV group than in the SARS-CoV-2 group. Antibiotics were more commonly administered in HCoVs than SARS-CoV-2 infection (42.1% vs. 100%, p<0.001). In the endemic HCoV group, 15% of the patients died. There were no significant differences in oxygen therapy, mechanical ventilation, and renal replacement therapy between the two groups.

**Table 1:** Baseline characteristics of the patients.

Variables	Total (n = 59)	SARS-CoV-2 pneumonia (n = 19)	Endemic HCoVs pneumonia (n = 40)	*P*-value
Age (years)	70 (62–78)	71 (60–66)	75 (64–78)	0.123
Male, n (%)	32 (54.2)	11 (57.9)	21 (52.5)	0.698
Body mass index (kg/m^2^)	23.4 (20.5–25.2)	24.0 (22.2–24.4)	23.4 (19.8–25.9)	0.395
Current or former smoker, n (%)	21 (35.6)	7 (36.8)	14 (35)	0.890
Symptom onset to hospital admission (days)	3 (1–7)	7 (3–8)	2 (0–5)	0.025
Symptoms and signs, n (%)				
Fever	52 (88.1)	18 (94.7)	34 (85.0)	0.411
Cough	30 (50.8)	14 (73.7)	16 (40.0)	0.014
Sputum	24 (40.7)	8 (42.1)	16 (40.0)	0.878
Sore throat	10 (16.9)	9 (47.4)	1 (2.5)	<0.001
Dyspnea	22 (37.3)	3 (15.8)	19 (47.5)	0.019
Headache	3 (5.1)	3 (15.8)	0 (0)	0.030
Myalgia	10 (16.9)	10 (52.6)	0 (0)	<0.001
Hyposmia	1 (1.7)	1 (5.3)	0 (0)	0.322
Other	4 (6.8)	0 (0)	4 (10.0)	0.294
Duration of fever (days)	2 (1–5)	4 (3–6)	2 (1–4)	0.016
Comorbidities, n (%)	47 (79.7)	15 (78.9)	32 (80.0)	1.000
Hypertension	27 (45.8)	10 (52.6)	17 (42.5)	0.465
Diabetes mellitus	16 (27.1)	4 (21.1)	12 (30.0)	0.470
Chronic lung disease	15 (25.4)	2 (10.5)	13 (32.5)	0.110
Chronic kidney disease	6 (10.2)	0 (0)	6 (15)	0.163
Cardiovascular disease	4 (6.8)	0 (0)	4 (10.0)	0.294
Cerebrovascular disease	13 (22.0)	0 (0)	13 (32.5)	0.005
Malignancy	10 (16.9)	2 (10.5)	8 (20.0)	0.476
CCI	4 (2–5)	3 (2–4)	4 (3–5)	0.002
CURB-65	1 (1–2)	1 (0–1)	2 (1–2)	0.001
MEWS	3 (2–4)	2 (2–3)	3 (2–4)	0.020
SOFA score	1 (0–3)	0 (0–2)	2 (0–4)	0.020
Vital signs at admission				
Systolic blood pressure	120 (110–140)	129 (122–138)	119 (110–140)	0.133
Diastolic blood pressure	70 (60–80)	77 (67–86)	70 (60–80)	0.083
Heart rate	90 (80–102)	88 (80–96)	92 (80–102)	0.338
Body temperature	37.0 (36.6–37.8)	37.6 (37.0–38.1)	36.9 (36.5–37.5)	0.024
Treatment, n (%)				
Antibiotics	48 (81.4)	8 (42.1)	40 (100)	<0.001
Corticosteroid	18 (30.5)	1 (5.3)	17 (42.5)	0.004
Oxygen therapy	37 (62.7)	10 (52.6)	27 (67.5)	0.270
Vasopressor	12 (20.3)	2 (10.5)	10 (25.0)	0.303
Mechanical ventilation	15 (25.4)	2 (10.5)	13 (32.5)	0.110
Renal replacement therapy	3 (5.1)	0 (0.0)	3 (7.5)	0.544
Length of stay(days)	16 (11–23)	17 (16–20)	15 (8–25)	0.267
In-hospital death, n (%)	6 (10.2)	0 (0.0)	6 (15.0)	0.163

Data are shown as the median (IQR) or number (%) as appropriate.

SARS-CoV-2, severe acute respiratory syndrome coronavirus 2; HCoVs, human coronaviruses; CCI, Charlson comorbidity index; CURB-65, Confusion, Urea, Respiratory rate, Blood pressure plus age ≥ 65 years; MEWS, modified early warning score; SOFA score, sequential organ failure assessment score.

The laboratory findings are shown in Table 2. White blood cell and neutrophil counts were higher in the endemic HCoV group than in the SARS-CoV-2 group (4.3 ×10^9^/L [IQR, 3.7–5.8] vs. 10.3 ×10^9^/L [IQR, 6.2.13.0], p<0.001; and 3.0 ×10^9^/L [IQR, 2.2–4.3] vs. 8.4 ×10^9^/L [IQR, 4.9–11.6], p<0.001, respectively). Liver enzymes such as aspartate transaminase and alanine transaminase were higher in the SARS-CoV-2 group than in the endemic HCoV group. Lactate dehydrogenase (LDH) was higher in the SARS-CoV-2 group (316 IU/L [IQR, 220–396] vs. 255 IU/L [IQR, 186–286], p = 0.042) and C-reactive protein (CRP) was higher in the endemic HCoV group (29.5 mg/L [IQR, 11.8–163.3] vs. 101 mg/L [IQR, 41.6–184.2], p<0.001).

**Table 2:** Laboratory findings and chest imaging of the patients.

Variables	Total (n = 59)	SARS-CoV-2 pneumonia (n = 19)	Endemic HCoVs pneumonia (n = 40)	*P*-value
Blood routine test				
White blood cells (×10^9^/L)	7.9 (4.1–11.5)	4.3 (3.7–5.8)	10.1 (6.2–13.0)	<0.001
Neutrophils (×10^9^/L)	6.5 (2.8–9.2)	3.0 (2.2–4.3)	8.4 (4.9–11.6)	<0.001
Lymphocytes (×10^9^/L)	0.9 (0.7–1.3)	8.3 (7.0–11.9)	9.0 (7.0–12.8)	0.789
Neutrophil-lymphocyte ratio	6.6 (3.1–10.3)	3.6 (2.3–6.6)	8.3 (4.5–16.3)	0.001
Hemoglobin (g/dL)	12.5 (10.2–13.3)	13.2 (12.8–15.0)	11.6 (9.9–12.8)	<0.001
RDW (%)	13.2 (12.2–14.3)	12. 4(12.1–12.9)	13.8 (12.7–14.9)	0.001
Platelets (×10^9^/L)	206 (154–290)	173 (150–227)	211 (162–313)	0.110
Biochemical test				
Total bilirubin (mg/dL)	0.5 (0.3–0.6)	0.6 (0.4–0.6)	0.5 (0.3–0.6)	0.285
Albumin (g/dL)	3.7 (3.2–4.2)	4.2 (3.8–4.4)	3.6 (3.1–3.8)	<0.001
AST (IU/L)	31 (21–45)	39 (31–81)	26 (20–40)	0.007
ALT (IU/L)	22 (13–44)	26 (21–51)	19 (10–30)	0.036
LDH (IU/L)	257 (202–335)	316 (220–396)	255 (186–286)	0.042
BUN (mg/dL)	16 (12–23)	12 (11.5–17)	19 (14–28)	0.005
Creatinine (mg/dL)	0.80 (0.62–1.12)	0.78 (0.59–0.88)	0.82 (0.64–1.26)	0.314
Sodium (mEq/L)	136 (134–138)	136 (135–139)	136 (134–137)	0.372
Potassium (mEq/L)	4.0 (3.8–4.2)	3.9 (3.8–4.2)	4.1 (3.7–4.2)	0.415
CRP (mg/L)	77.8 (26.9–181.8)	29.5 (11.8–163.3)	101 (41.6–184.2)	<0.001

Data are shown as the median (IQR) or number (%) as appropriate.

SARS-CoV-2, severe acute respiratory syndrome coronavirus 2; HCoV, human coronavirus; RDW, red cell distribution width; AST, aspartate transaminase; ALT, alanine transaminase; LDH, lactate dehydrogenase; BUN, blood urea nitrogen; CRP, C-reactive protein.

**Fig 2:**
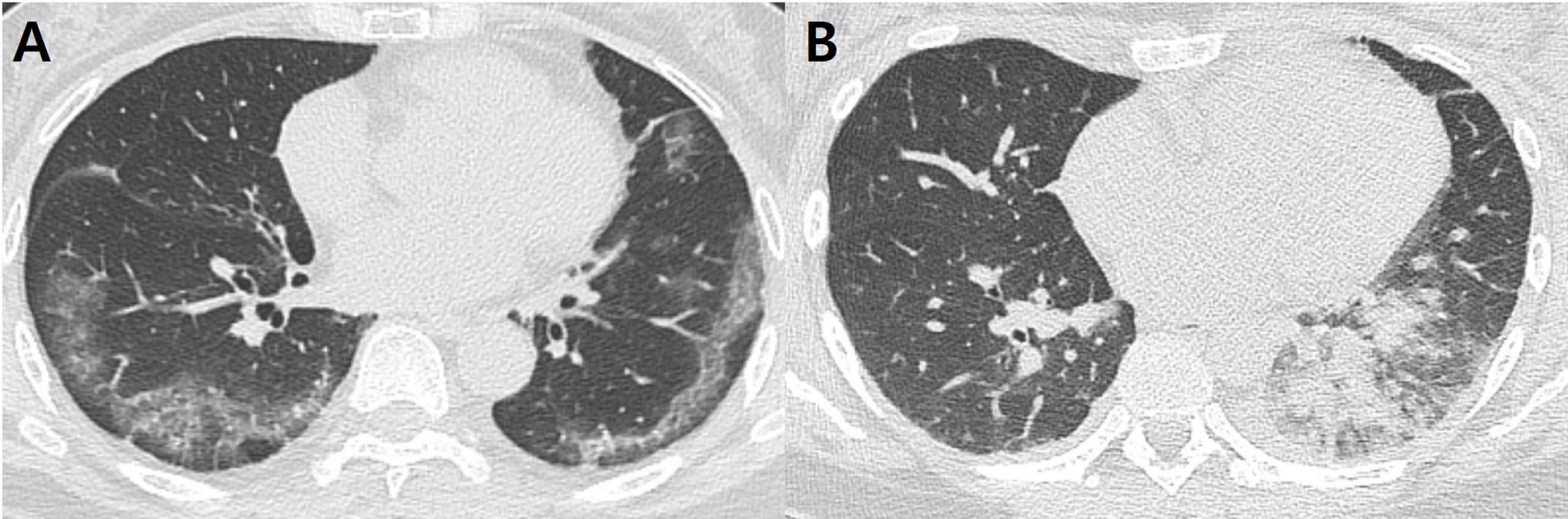
Representative cases of SARS-CoV-2 (A) and endemic HCoVs (B) pneumonia. (A) Pneumonia due to SARS-CoV-2 in a 63-year-old female. Axial computed tomography (CT) shows multifocal ground-glass opacities (GGOs), predominantly located in the peripheral areas of both lungs. (B) Pneumonia due to HCoV-229E in a 35-year-old female. Axial CT shows patchy and nodular consolidation and GGO in the left lower lobe. A small amount of pleural effusion is also present.

A comparison of the radiographic findings between SARS-CoV-2 and endemic HCoV pneumonia is summarized in Table 3. All 59 patients underwent CXRs, and chest CT was performed in 54 patients. Among them, two patients with end-stage idiopathic pulmonary fibrosis and severe lung destruction due to non-tuberculous mycobacterial infection were excluded from the imaging analysis. Thus, CXRs from 57 patients and CT scans from 52 patients were evaluated. Initial CXRs were acquired on admission, and CT scans were acquired within a week from admission (median, 0; range, 0–7 days). Although there was no statistically significant difference, diffuse opacity was more prevalent in patients with SARS-CoV-2 pneumonia (12/18; 66.7%) than in those with endemic HCoV pneumonia (22/39; 56.4%). In terms of serial changes, most of the patients with SARS-CoV-2 pneumonia showed improvement at the end (Type I and II; 17/18; 94.4%), and none showed radiographic deterioration. In contrast, 10.3% (Type IV; 4/39) of the patients with endemic HCoV pneumonia showed progressive deterioration of the CXR findings. Regarding the CT findings, patients with SARS-CoV-2 demonstrated a significantly higher rate of bilateral pneumonia (88.9% vs. 58.8%; p = 0.024) and a greater number of involved lobes (4.11 ± 1.32 vs. 3.09 ± 1.51; p = 0.026), compared to those with endemic HCoV pneumonia. However, the difference in the total CT score between the two groups was not significant (9.11 ± 6.04 vs. 7.85 ± 5.62; p = 0.434). The most frequent CT pattern in SARS-CoV-2 pneumonia was GGO only (83.3%), whereas mixed pattern (GGO + consolidation) was predominant in endemic HCoV pneumonia (64.7%) (p < 0.001). In terms of anatomic location, the rate of peripheral predominance was significantly higher in patients with SARS-CoV-2 than in those with endemic HCoV pneumonia (72.2% vs. 35.3%; p = 0.019). The presence of micronodules (35.3% vs. 0%; p = 0.004) and pleural effusion (47.1% vs. 5.6%; p = 0.002) were significantly more prevalent in endemic HCoV pneumonia than in SARS-CoV-2 (Fig 2). Excellent interobserver agreements were noted for the total CT scores (ICC = 0.946, p < 0.001).

**Table 3:** Imaging findings of the patients.

Variables	Total	SARS-CoV-2 pneumonia	Endemic HCoVs pneumonia	*P*-value
Chest X-ray	(n = 57)	(n = 18)	(n = 39)	
Lesion extent				0.261
Normal	6 (10.5)	3 (16.7)	3 (7.7)	
Focal	17 (29.8)	3 (16.7)	14 (35.9)	
Diffuse	34 (59.7)	12 (66.7)	22 (56.4)	
Serial change^a^				0.431
Type I	35 (61.4)	11 (61.1)	24 (61.5)	
Type II	14 (24.6)	6 (33.3)	8 (20.5)	
Type III	4 (7.0)	1 (5.6)	3 (7.7)	
Type IV	4 (7.0)	0 (0)	4 (10.3)	
Chest CT findings	(n = 52)	(n = 18)	(n = 34)	
Laterality				0.024
Unilateral pneumonia	16 (30.8)	2 (11.1)	14 (41.2)	
Bilateral pneumonia	36 (69.2)	16 (88.9)	20 (58.8)	
Number of involved lobes^b^	3.44 ± 1.51	4.11 ± 1.32	3.09 ± 1.51	0.026
Pattern				<0.001
GGO only	23 (44.2)	15 (83.3)	8 (23.5)	
Consolidation	4 (7.7)	0 (0)	4 (11.8)	
Both GGO and consolidation	25 (48.1)	3 (16.7)	22 (64.7)	
Presence of micronodules	12 (23.1)	0 (0)	12 (35.3)	0.004
Longitudinal distribution				0.286
Upper predominance	4 (7.7)	0 (0)	4 (11.8)	
Lower predominance	26 (50.0)	9 (50.0)	17 (50.0)	
Random	22 (42.3)	9 (50.0)	13 (38.2)	
Axial distribution				0.019
Central predominance	0 (0)	0 (0)	0 (0)	
Peripheral predominance	25 (48.1)	13 (72.2)	12 (35.3)	
Mixed	27 (51.9)	5 (27.8)	22 (64.7)	
Presence of pleural effusion	17 (29.8)	1 (5.6)	16 (47.1)	0.002
Presence of lymphadenopathy	2 (3.8)	1 (5.6)	1 (2.9)	0.999
Total CT score ^b^	8.29 ± 5.74	9.11 ± 6.04	7.85 ± 5.62	0.434

Data are shown as the number (%) or mean ± standard deviation, as appropriate.

^a^Serial changes in the chest X-rays were classified as follows: type 1, radiographic improvement; type 2, radiographic deterioration by one peak level followed by improvement; type 3, fluctuating radiographic changes with at least two peaks; and type 4, progressive radiographic deterioration.

^b^Average of the number of involved lobes and CT score.

SARS-CoV-2, severe acute respiratory syndrome coronavirus 2; HCoVs, human coronaviruses; CT, computed tomography; GGO, ground-glass opacity.

The coinfection status is presented in Table 4. No pathogen co-infected with SARS-CoV-2 pneumonia was identified, whereas 55% of endemic HCoV pneumonia cases had co-infected pathogens. The most common co-infected pathogens were *Streptococcus pneumoniae* (12.5%), *Haemophilus influenza* (12.5%), and influenza virus (10%).

**Table 4:** Coinfection status of the patients.

Pathogens	SARS-CoV-2 pneumonia (n = 19)	Endemic HCoVs pneumonia			
		Overall (n = 40)	HCoV-229E (n = 15)	HCoV-OC43 (n = 21)	HCoV-NL63 (n = 6)
Overall	0 (0)	22 (55)	6 (40)	11 (52.4)	5 (83.3)
2 or more	0 (0)	9 (22.5)	3 (20)	1 (4.8)	5 (83.3)
Bacteria	0 (0)	15 (37.5)	4 (26.7)	7 (33.3)	4 (66.7)
*Haemophilus influenzae*	0 (0)	5 (12.5)	0 (0)	1 (4.8)	4 (66.7)
*Streptococcus pneumoniae*	0 (0)	5 (12.5)	0 (0)	1 (4.8)	4 (66.7)
*Mycoplasma pneumoniae*	0 (0)	3 (7.5)	0 (0)	3 (14.3)	0 (0)
*Acinetobacter baumannii*	0 (0)	3 (7.5)	1 (6.7)	1 (4.8)	1 (16.7)
*Klebsiella pneumoniae*	0 (0)	2 (5.0)	1 (6.7)	1 (4.8)	0 (0)
*Staphylococcus aureus*	0 (0)	1 (2.5)	1 (6.7)	0 (0)	0 (0)
*Chlamydia pneumoniae*	0 (0)	1 (2.5)	1 (6.7)	0 (0)	0 (0)
*Legionella pneumophila*	0 (0)	1 (2.5)	0 (0)	1 (4.8)	0 (0)
*Escherichia coli*	0 (0)	1 (2.5)	0 (0)	1 (4.8)	0 (0)
*Pseudomonas aeruginosa*	0 (0)	1 (2.5)	1 (6.7)	0 (0)	0 (0)
Virus	0 (0)	5 (12.5)	2 (13.3)	1 (4.8)	2 (33.3)
Influenza virus	0 (0)	4 (10)	2 (13.3)	1 (4.8)	1 (16.7)
Rhinovirus	0 (0)	1 (2.5)	1 (6.7)	0 (0)	0 (0)
Respiratory syncytial virus	0 (0)	1 (2.5)	0 (0)	0 (0)	1 (16.7)
Other					
*Pneumocystis jirovecii*	0 (0)	3 (7.5)	2 (13.3)	0 (0)	1 (16.7)

Data are shown as a number (%).

SARS-CoV-2, severe acute respiratory syndrome coronavirus 2; HCoVs, human coronaviruses; NA, not applicable.

## Discussion

In this study, findings of adult hospitalized patients with SARS-CoV-2 CAP were compared with those with endemic HCoV CAP. Cough, sore throat, headache, myalgia, and prolonged fever were more commonly observed in patients with SARS-CoV-2, but dyspnea, leukocytosis with left shift, and increased CRP were more common in patients with endemic HCoVs. Involvement of both lungs, multi-lobar involvement, GGO, and peripheral involvement were more frequently found in the chest radiographs of the SARS-CoV-2 group, whereas micronodules and pleural effusion were more frequent in the endemic HCoV group. Although more than half of the patients in the endemic HCoV group were co-infected with other pathogens, patients in the SARS-CoV-2 group were not coinfected.

In our study population, patients with SARS-CoV-2 pneumonia presented with influenza-like illnesses such as sore throat, cough, myalgia, and fever as initial symptoms. In contrast, patients with endemic HCoV pneumonia presented with bacterial pneumonia, dyspnea, leukocytosis with left shift, and increased CRP at admission. There were no co-infected pathogens in SARS-CoV-2 pneumonia, but bacterial pathogens were simultaneously detected in 38% of endemic HCoV pneumonia cases. Therefore, it is suggested that the difference in the frequency of bacterial coinfection may be one of the causes of the differences in clinical presentation between the two groups. Another possible cause is the difference in virulence between endemic HCoVs and SARS-CoV-2. While endemic HCoVs usually cause common cold and infrequently pneumonia, SARS-CoV-2 appears to cause pneumonia easily during this pandemic situation. Thus, SARS-CoV-2 is thought to be more virulent than endemic HCoVs. If this is true, it is suggested that SARS-CoV-2 causes pneumonia easily without further bacterial infection, whereas endemic HCoVs cause pneumonia mainly through further bacterial infection [16]. This distinction may be responsible for the above differences in clinical aspects. Furthermore, Gussow et al. reported that the spike protein of SARS-CoV-2 has a structure that can more strongly bind to human cells than that of endemic HCoVs [17]. The novelty of SARS-CoV-2 may be another cause of these differences. This suggests that the absence of pre-existing immunity against SARS-CoV-2 may cause more active viral replication and subsequent inflammation in SARS-CoV-2 CAP than in endemic HCoV CAP. Further research is required to determine why differences in clinical features arise. Such a study may be helpful in speculating whether SARS-CoV-2 infection will change to seasonal viral diseases such as endemic HCoV infection, just as the 2009 pandemic influenza A infection has turned to seasonal influenza.

The characteristic radiologic findings of SARS-CoV-2 infection were bilateral and peripheral GGOs and consolidation [18]. It has been reported that more than 70% of SARS-CoV-2 pneumonia cases exhibit typical CT findings [19]. Indeed, a study on the performance of radiologists in differentiating SARS-CoV-2 pneumonia from other types of viral pneumonia based on CT findings found that SARS-CoV-2 pneumonia could be correctly distinguished with 67%-97% sensitivity [20]. These findings support our hypothesis that SARS-CoV-2 with relatively severe virulence causes pneumonia by directly involving the lower respiratory tract. Consistent with the findings of previous studies, our study results demonstrate that bilateral pneumonia and GGO with peripheral distribution were discriminating features of SARS-CoV-2 pneumonia compared with endemic HCoV pneumonia. In contrast to the rare incidence of pleural effusion in SARS-CoV-2 pneumonia (one of 18 cases), 47.1% of the endemic HCoV group presented pleural effusion in our study, which is far higher than the reported incidence of pleural effusion in viral pneumonia [18, 21]. The discrepancy in the incidence of pleural effusion could be due to various comorbidities of the endemic HCoV pneumonia group, such as cardiac diseases, renal diseases, and malignancy. In addition, the prevalence of coinfection in patients with endemic HCoV pneumonia might also be one cause of pleural effusion.

This study has several limitations. First, because it was a retrospective study in a single center, only a small number of patients were included. However, this is the first study to compare the clinical features of SARS-CoV-2 CAP with those of endemic HCoV CAP. Second, in the SARS-CoV-2 group, several patients with mild symptoms were hospitalized at the early stage of the outbreak for isolation purposes. Compared to studies that included a large study population, our study had a lower mortality rate of SARS-CoV-2 infection [22, 23]. In the case of endemic HCoVs, only patients who needed inpatient treatment were selected, so there may be differences in severity between the two groups. This could explain why the CCI, CURB-65, MEWS, and SOFA scores were higher in the patients with endemic HCoVs than in those with SARS-CoV-2. However, since both groups were selected only when they met the definition of CAP, the effect of this type of selection bias is not expected to be significant.

## Conclusions

Adult hospitalized patients with SARS-CoV-2 CAP had distinctive clinical and radiologic findings from those with endemic HCoV CAP. These differences led us to discover the unique properties of SARS-CoV-2 and shed light on the future progression of the COVID-19 pandemic.

## Supporting information

S1 Dataset (XLSX)

## References

[1] World Health O. Clinical management of severe acute respiratory infection when novel coronavirus (‎‎‎‎‎2019-nCoV)‎‎‎‎‎ infection is suspected: interim guidance, 28 January 2020. Geneva: World Health Organization, 2020 2020. Report No.: Contract No.: WHO/nCoV/Clinical/2020.3.

[2] Wiersinga WJ, Rhodes A, Cheng AC, Peacock SJ, Prescott HC. Pathophysiology, Transmission, Diagnosis, and Treatment of Coronavirus Disease 2019 (COVID-19): A Review. Jama. 2020;324(8): 782–793. 10.1001/jama.2020.12839 32648899

[3] Schnell D, Mayaux J, de Bazelaire C, Legoff J, Feuillet S, Scieux C, et al Risk factors for pneumonia in immunocompromised patients with influenza. Respiratory medicine. 2010;104(7): 1050–1056. 10.1016/j.rmed.2010.01.021 20181467

[4] Kanwar A, Selvaraju S, Esper F. Human Coronavirus-HKU1 Infection Among Adults in Cleveland, Ohio. Open forum infectious diseases. 2017;4(2): ofx052. 10.1093/ofid/ofx052 28616442 PMC5466428

[5] Yin Y, Wunderink RG. MERS, SARS and other coronaviruses as causes of pneumonia. Respirology (Carlton, Vic). 2018;23(2): 130–137. 10.1111/resp.13196 29052924 PMC7169239

[6] Galante O, Avni YS, Fuchs L, Ferster OA, Almog Y. Coronavirus NL63-induced Adult Respiratory Distress Syndrome. American journal of respiratory and critical care medicine. 2016;193(1): 100–101. 10.1164/rccm.201506-1239LE 26720790

[7] Pene F, Merlat A, Vabret A, Rozenberg F, Buzyn A, Dreyfus F, et al Coronavirus 229E-related pneumonia in immunocompromised patients. Clin Infect Dis. 2003;37(7): 929–932. 10.1086/377612 13130404 PMC7107892

[8] Walsh EE, Shin JH, Falsey AR. Clinical impact of human coronaviruses 229E and OC43 infection in diverse adult populations. The Journal of infectious diseases. 2013;208(10): 1634–1642. 10.1093/infdis/jit393 23922367 PMC3805243

[9] Kaur N, Singh R, Dar Z, Bijarnia RK, Dhingra N, Kaur T. Genetic comparison among various coronavirus strains for the identification of potential vaccine targets of SARS-CoV2. Infection, genetics and evolution: journal of molecular epidemiology and evolutionary genetics in infectious diseases. 2020 10.1016/j.meegid.2020.104490 32745811 PMC7395230

[10] Hong KH, Lee SW, Kim TS, Huh HJ, Lee J, Kim SY, et al Guidelines for Laboratory Diagnosis of Coronavirus Disease 2019 (COVID-19) in Korea. Annals of laboratory medicine. 2020;40(5): 351–360. 10.3343/alm.2020.40.5.351 32237288 PMC7169629

[11] Mandell LA, Wunderink RG, Anzueto A, Bartlett JG, Campbell GD, Dean NC, et al Infectious Diseases Society of America/American Thoracic Society consensus guidelines on the management of community-acquired pneumonia in adults. Clin Infect Dis. 2007;44 Suppl 2(Suppl 2): S27–72. 10.1086/511159 17278083 PMC7107997

[12] Das KM, Lee EY, Enani MA, AlJawder SE, Singh R, Bashir S, et al CT correlation with outcomes in 15 patients with acute Middle East respiratory syndrome coronavirus. AJR American journal of roentgenology. 2015;204(4): 736–742. 10.2214/AJR.14.13671 25615627

[13] Wong KT, Antonio GE, Hui DS, Lee N, Yuen EH, Wu A, et al Thin-section CT of severe acute respiratory syndrome: evaluation of 73 patients exposed to or with the disease. Radiology. 2003;228(2): 395–400. 10.1148/radiol.2283030541 12738877

[14] Hansell DM, Bankier AA, MacMahon H, McLoud TC, Müller NL, Remy J. Fleischner Society: glossary of terms for thoracic imaging. Radiology. 2008;246(3): 697–722. 10.1148/radiol.2462070712 18195376

[15] Ooi GC, Khong PL, Müller NL, Yiu WC, Zhou LJ, Ho JC, et al Severe acute respiratory syndrome: temporal lung changes at thin-section CT in 30 patients. Radiology. 2004;230(3): 836–844. 10.1148/radiol.2303030853 14990845

[16] Subbarao K, Mahanty S. Respiratory Virus Infections: Understanding COVID-19. Immunity. 2020;52(6): 905–909. 10.1016/j.immuni.2020.05.004 32497522 PMC7237932

[17] Gussow AB, Auslander N, Faure G, Wolf YI, Zhang F, Koonin EV. Genomic determinants of pathogenicity in SARS-CoV-2 and other human coronaviruses. Proceedings of the National Academy of Sciences of the United States of America. 2020;117(26): 15193–15199. 10.1073/pnas.2008176117 32522874 PMC7334499

[18] Bernheim A, Mei X, Huang M, Yang Y, Fayad ZA, Zhang N, et al Chest CT Findings in Coronavirus Disease-19 (COVID-19): Relationship to Duration of Infection. Radiology. 2020;295(3): 200463 10.1148/radiol.2020200463 32077789 PMC7233369

[19] Koo HJ, Choi SH, Sung H, Choe J, Do KH. RadioGraphics Update: Radiographic and CT Features of Viral Pneumonia. Radiographics: a review publication of the Radiological Society of North America, Inc. 2020;40(4): E8–e15. 10.1148/rg.2020200097 32501740 PMC7336757

[20] Bai HX, Hsieh B, Xiong Z, Halsey K, Choi JW, Tran TML, et al Performance of Radiologists in Differentiating COVID-19 from Non-COVID-19 Viral Pneumonia at Chest CT. Radiology. 2020;296(2): E46–e54. 10.1148/radiol.2020200823 32155105 PMC7233414

[21] Shi H, Han X, Jiang N, Cao Y, Alwalid O, Gu J, et al Radiological findings from 81 patients with COVID-19 pneumonia in Wuhan, China: a descriptive study. The Lancet Infectious diseases. 2020;20(4): 425–434. 10.1016/S1473-3099(20)30086-4 32105637 PMC7159053

[22] RAAS inhibitors are not associated with mortality in COVID-19 patients: Findings from an observational multicenter study in Italy and a meta-analysis of 19 studies. Vascular pharmacology. 2020;135: 106805 10.1016/j.vph.2020.106805 32992048 PMC7521934

[23] Di Castelnuovo A, Bonaccio M, Costanzo S, Gialluisi A, Antinori A, Berselli N, et al Common cardiovascular risk factors and in-hospital mortality in 3,894 patients with COVID-19: survival analysis and machine learning-based findings from the multicentre Italian CORIST Study. Nutrition, metabolism, and cardiovascular diseases: NMCD. 2020;30(11): 1899–1913. 10.1016/j.numecd.2020.07.031 32912793 PMC7833278

